# Positive correlation between serum and peritoneal fluid CA-125 levels in women with pelvic endometriosis

**DOI:** 10.1590/S1516-31802006000400010

**Published:** 2006-05-04

**Authors:** Vivian Ferreira do Amaral, Rui Alberto Ferriani, Marcos Felipe Silva de Sá, Antonio Alberto Nogueira, Julio César Rosa e Silva, Ana Carolina Japur de Sá Rosa e Silva, Marcos Dias de Moura

**Keywords:** Endometriosis, CA-125 antigen, Estradiol, Laparoscopy, Infertility, Endometriose, Antígeno CA-125, Estradiol, Laparoscopia, Infertilidade

## Abstract

**CONTEXT AND OBJECTIVE:**

One of the diagnostic markers of endometriosis is CA-125, and elevated levels of this are caused by high concentrations in the ectopic endometrium. The objective of this study was to correlate CA-125 levels in serum and peritoneal fluid from women with and without pelvic endometriosis.

**DESIGN AND SETTING:**

This was a prospective, cross-sectional, controlled study of consecutive pa tients undergoing laparoscopy for infertility, pelvic pain or tubal ligation, during early follicular phase, at the university hospital of Faculdade de Medicina de Ribeirão Preto.

**METHODS:**

Fifty-two patients were divided into two groups: endometriosis group, consisting of 35 patients with biopsy-confirmed pelvic endometriosis, and control group, consisting of 17 patients without endometriosis. CA-125 levels in serum samples and peritoneal fluid were determined by chemiluminescence.

**RESULTS:**

CA-125 levels in serum and peritoneal fluid were higher in patients with advanced pelvic endometriosis (means of 39.1 ± 45.8 U/ml versus 10.5 ± 5.9 U/ml in serum, p < 0.005; 1,469.4 ± 1,350.4 U/ml versus 888.7 ± 784.3 U/ml in peritoneal fluid, p < 0.05), and showed a positive correlation between each other (correlation coefficient (r) = 0.4880). Women with more advanced degrees of endometriosis showed higher CA-125 levels in both serum and peritoneal fluid (p = 0.0001).

**CONCLUSION:**

There is a positive correlation between serum and peritoneal fluid values of CA-125 in women with and without endometriosis, and their levels are higher in peritoneal fluid. Advanced endometriosis is related to higher levels in both serum and peritoneal fluid.

## Resumo

**CONTEXTO E OBJETIVO:**

Um dos marcadores diagnósticos de endometriose é o CA-125, e seus níveis elevados são devidos à alta concentração no endométrio ectópico. O objetivo deste estudo foi correlacionar os níveis de CA-125 no soro e fluido peritonial de mulheres com e sem endometriose pélvica.

**TIPO DE ESTUDO E LOCAL:**

Estudo prospectivo, longitudinal, controlado, de pacientes consecutivas submetidas a laparoscopia por infertilidade, dor pélvica ou laqueadura tubária, durante a fase folicular precoce no Hospital Universitário da Faculdade de Medicina de Ribeirão Preto.

**MÉTODOS:**

Cinqüenta e duas pacientes foram divididas em dois grupos: grupo endometriose, com 35 pacientes com biópsia confirmada de endometriose pélvica, e grupo controle, com 17 pacientes sem endometriose. Níveis de CA-125 em amostras no soro e fluido peritonial foram determinadas por quimiluminescência.

**RESULTADOS:**

Os níveis de CA-125 no soro e fluido peritonial foram mais altos nas pacientes com endometriose pélvica avançada (média 39,1 ± 45,8 U/ml *versus* 10,5 ± 5,9 U/ml no soro, p < 0,005, 1469,4 ± 1350,4 U/ml *versus* 888,7 ± 784,3 U/ml no fluido peritonial, p < 0,05), e o estudo mostrou uma correlação positiva entre eles (coeficiente de correlação = 0,4880). Mulheres com estágios mais avançados de endometriose mostraram níveis de CA-125 maiores em ambos soro e fluido peritonial (p = 0,0001).

**CONCLUSÃO:**

Há uma correlação positiva entre os valores de CA-125 no soro e no fluido peritonial em pacientes com e sem endometriose e seus níveis são maiores no fluido peritonial. Endometriose avançada é relacionada com níveis mais altos de CA-125 em ambos soro e fluido peritonial.

## INTRODUCTION

Endometriosis is characterized by the presence of endometrial tissue, consisting of glands and/or stroma located outside the uterus. The incidence of the disorder in infertile patients ranges from 10 to 25%, with prevalence increasing to 60 to 70% in cases of chronic pelvic pain, and it may be present in 1 to 20% of asymptomatic women.^1^

One of the diagnostic markers already well established is CA-125, a high molecular weight glycoprotein (> 20,000 Da) of epithelial origin found in normal cells,^2,3^ and which is produced in the celomic epithelium during embryonic development.^4^ The OC-125 marker, a monoclonal antibody that recognizes an antigenic determinant in CA-125, was developed thereafter. In adult tissues, CA-125 has been detected in normal and neoplastic epithelium of celomic origin such as endometrium, endocervix, epithelial cells of Fallopian tubes and cancerous epithelial cells of the ovary. The first study of this marker in patients with endometriosis detected elevated serum levels.^5^ The elevation of this marker in women with endometriosis is due to its higher concentration in ectopic than in entopic endometrium. This increase is also due to inflammatory reactions, which alter endothelial permeability, thereby allowing the marker to reach the circulation.^2^

Koninckx & Martin, in a study of CA-125 levels in patients with pelvic endometriosis^6^ concluded that superficial disease leads to a greater increase in this marker in peritoneal fluid, whereas in deep disease CA-125 levels are higher in blood and lower in peritoneal fluid. Abrão et al. evaluated serum CA-125, C-reactive protein, amyloid A protein and anticardiolipin antibodies during the menstrual phase and the middle follicular phase, and concluded that CA-125 was the marker found to be present at highest levels in patients with advanced endometriosis when measured during the menstrual phase.^1^

Peritoneal fluid CA-125 levels are higher than serum CA-125, but no significant difference is observed between the peritoneal fluid levels of CA-125 in women with and without endometriosis^7,8^ Levels of CA-125 in peritoneal fluid seem to be a more sensitive indicator of disease than the level in serum, and the measurement of CA-125 in peritoneal fluid could be useful in the detection of early-stage endometriosis.^9–11^ The limited number of studies combining peritoneal and serum samples and the small number of cases studied encouraged us to reevaluate the concentrations of CA-125 in peritoneal fluid and serum in women with and without endometriosis.

## OBJECTIVE

The objective of the present study was to correlate CA-125 and 17β-estradiol (E2) levels in serum and peritoneal fluid from women with and without pelvic endometriosis.

## MATERIALS AND METHODS

### Study design Study design

This was a prospective cross-sectional study.

### Setting

The study was conducted in the university hospital of Faculdade de Medicina de Ribeirão Preto, Universidade de São Paulo, which is a tertiary-level public hospital.

### Subjects

Fifty-two consecutive women who underwent laparoscopy during the early follicular phase (because of clinical indication of infertility, pelvic pain or tubal ligation) were selected and divided into two groups. The endometriosis group consisted of 35 women with pelvic endometriosis confirmed by biopsy and the control group consisted of 17 women without pelvic endometriosis. The exclusion criteria were the use of hormonal medications during the six months prior to the laparos copy, presentation of ovarian neoplasia or pelvic inflammatory disease as an intraoperative finding, pregnancy, or refusal to participate.

The study was approved by the Research Ethics Committee of the university hospital, Faculdade de Medicina de Ribeirão Preto, Universidade de São Paulo, and all patients gave informed written consent to participate in the project.

### Blood and peritoneal fluid collection

Blood samples were collected in the early follicular phase during the laparoscopy procedure, placed in dry test tubes, identified and left to stand for 30 minutes to three hours, or until blood clotting occurred. Peritoneal fluid was aspirated from the anterior uterovesical space and from the posterior *cul-de-sac* during laparoscopy. The volume of peritoneal fluid was noted. The peritoneal fluid sample was placed in a sterile tube and centrifuged at 1,500 g for 10 min. The supernatant was collected and stored at −18° C until assayed. In women with pelvic endometriosis, the disease was staged according to the classification of the American Society for Reproductive Medicine, as revised in 1996.

### Laboratory analysis

CA-125 was measured using an OM-MA Immulite 2000 kit (DPC; Diagnostic Products Corporation, Los Angeles, California, United States) by the chemiluminescence method in duplicate. All samples underwent analysis using the same kit in a single assay. The sensitivity of the CA-125 test is 1 U/ml, with high specificity for CA-125 and low cross-reaction with CA15-3 (0.41%) and CEA (0.05%); the intra-assay error was 4.03%.

### Statistical analysis Statistical analysis

Data were analyzed statistically using the Stata software, with the level of significance set at p < 0.05. The Kruskal-Wallis test was used for nonparametric variables, analysis of variance (ANOVA) was used in the presence of two or more variables, and the Fisher test was used when the expected proportion was less than five.

## RESULTS

The mean age was 29.2 ± 5.6 years for the endometriosis group and 34.0 ± 3.2 years for the control group (p > 0.01). The major complaints reported by the 35 women with pelvic endometriosis were dysmenorrhea (91.0%), infertility (71.4%) and dyspareunia (57.1%). There were no clinical complaints in the control group.

In the women with pelvic endometriosis, serum CA-125 levels ranged from 8.3 to 115.3 U/ml, with a mean of 39.1 ± 45.8 U/ml. In the control women, serum CA-125 levels were significantly lower (p = 0.005), ranging from 2.7 to 25.0 U/ml, with a mean of 10.5 ± 5.9 U/ml. In the endometriosis group, peritoneal fluid CA-125 levels ranged from 268.0 to 7,615.0 U/ml, with a mean of 1,469.4 ± 1,350.4 U/ml, while in the control group, peritoneal fluid CA-125 levels were significantly lower than in the endometriosis group (p = 0.028), ranging from 221.0 to 2,345.0 U/ml, with a mean of 888.7 ± 784.3 U/ml.

**Figure 1:**
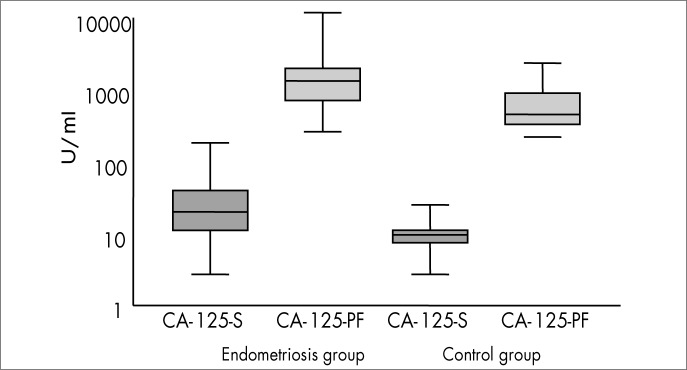
Mean CA-125 levels in serum (S) and peritoneal fluid (PF) from women with and without pelvic endometriosis.

**Figure 2:**
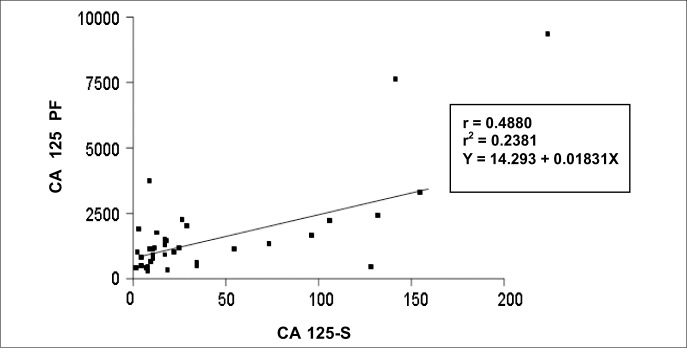
Correlation between CA-125 levels in serum (S) and peritoneal fluid (PF) from women with pelvic endometriosis.

Comparison of CA-125 values obtained for serum and peritoneal fluid in the endometriosis group showed a correlation between the two compartments, with a correlation coefficient (r) of 0.4880 and a coefficient of determination (r^2^) of 0.2381. When the tendency to inclination was tested, the result was statistically significant (p = 0.002). When linearity was tested by the equation Y = 14.293 + 0.01831X, nine samples were found to be above the line, 26 below the line and 14 on the same level (Figure 1). The linear trend of our sample was not significant (p = 0.49), as shown in Figure 2.

When the serum CA-125 levels of women with pelvic endometriosis were compared with disease staging, women with more advanced degrees of endometriosis showed higher CA-125 levels in both serum and peritoneal fluid (p = 0.0001). The analysis of peritoneal fluid volume demonstrated that the more advanced the disease was, the smaller the volume of peritoneal fluid detected at laparoscopy was (Table 1).

**Table 1.** Mean serum and peritoneal fluid CA-125 levels and staging of endometriosis in the endometriosis group, and range of peritoneal fluid volume compared with disease staging

Stage	Serum CA-125 (U/ml)*	Peritoneal fluid CA-125 (U/ml)†	Peritoneal fluid volume (ml)
I	8.3 ± 4.1	808.75 ± 505.5	10.0 - 15.0
II	14.8 ± 8.0	981.6 ± 547.7	7.5 - 12.0
III	37.8 ± 37.9	1,554.3 ± 871.8	5.0 - 10.0
IV	115.3 ± 35.4	2,817.0 ± 2,553.6	5.0 - 8.0

*Infergroup evaluation:*

* *p = 0.0001 and*

† *p = 0.03 (cutoff value for CA-15 = 16 U/ml).*

The lesions suggestive of endometriosis viewed during laparoscopy were divided into typical or pigmented (black, suggestive of chronic disease) and atypical or non-pigmented (red, suggesting active disease). There was no significant correlation between these lesions (typical and atypical) and serum or peritoneal fluid CA-125 levels (Table 2).

**Table 2.** Correlation between serum and peritoneal fluid CA-125 levels and type of endometriotic lesion viewed in laparoscopy, i.e. typical or pigmented (black) and atypical or non-pigmented (red)

CA-125 (U/ml)	Type of lesion		p
	Atypical (n = 16)	Typical (n = 19)	
Serum	22.5 ± 25.7	53.0 ± 54.4	> 0.05
Peritoneal fluid	1,627.2 ± 1,704.3	1,282.1 ± 760.8	> 0.05

## DISCUSSION

Increasing incidence of pelvic endometriosis has been observed over recent decades.^12^ Superficial endometriosis has been described as a cyclical and normal phenomenon in the life of a woman, with the development and evolution of this disease occurring in some women as a result of immunological alterations.^1^ Today, superficial endometriosis is thought to be a physiological and intermittent condition in women during their reproductive years, whereas evolving disease characterized as deep infiltrative endometriosis and endometrial ovarian cysts is considered to be the true disease.^12^

Divergences persist regarding the natural history of endometriosis, its symptoms, extent, location and staging.^13^ The incidence of pelvic pain, especially dysmenorrhea in women with endometriosis, plus infertility and dyspareunia, are the triad that characterize the disease. The severity of the disease and the intensity of symptoms presented by women with this disease are controversial subjects.^12^ In the present study, we observed the presence of dysmenorrhea as the major symptom, but none of the symptoms observed was correla ted with the severity of the disease, as also reported by Fedele et al.^14^ In our study, we also found no correlation between the number of endometriotic implants and severity of pelvic pain or dysmenorrhea and the disease staging according to the ASRM^15^ (not shown). The behavior of disease that solely develops at this site has characteristics that are distinct from peritoneal or rectovaginal septal disease.^16^

The low specificity of the diagnostic methods available, with respect to the severity of the disease, has motivated new studies. CA-125 has been described as one of the possible markers for endometriosis follow-up. The high levels of CA-125 in the blood stream observed in the presence of an endometriotic ovarian cyst and/or endometriosis with deep infiltration suggest that this antigen may pass into the circulation from endometrial cells of patients with endometriosis.^2,3,17^ The CA-125 released by the endometrium may reach the blood stream and lymphatic circulation by the peritoneal route, starting from retrograde menstruation, thereby allowing contact with local inflammatory reactions and thus releasing celomic CA-125.^17,18^ Another explanation for the increase in CA-125 in blood could be its access to the abdominal cavity through tubal reflux, resulting in absorption by peritoneal lymphatic vessels.^18^ Despite the mechanisms proposed, doubts still persist about the real mechanism for CA-125 release into the circulation, since retrograde menstruation is still controversial and the levels of this marker are altered during the postmenopausal period, as is the case for the utilization of CA-125 in the diagnosis of ovarian cancer.^18^

In the present study, evaluation of serum CA-125 levels showed that the mean levels of this marker in women with pelvic endometriosis were higher than in normal women, thus confirming data from other authors^19–21^ When calculating the correlation between CA-125 levels in serum and peritoneal fluid and the disease staging we noted a significant trend towards a linear increase in this marker with disease severity. Several investigators have detected this correlation between increased serum CA-125 levels and severity of the disease, thus indicating the diagnostic potential of this marker for patients with stage III and IV pelvic endometriosis.^2,11,22^ Some studies have detected higher CA-125 levels in the presence of stage I and II endometriosis,^23,24^ while others have reported increased CA-125 levels in stage III and IV endometriosis,^2,25,26^ as we also observed in our study.

The peritoneal fluid of women with endometriosis shows a more marked increase in macrophage numbers during the follicular phase.^27^ This increase causes higher local secretion of various products. Among these are growth factors and cytokines, which may be involved in the mechanism for the implantation and subsequent development and proliferation of endometriotic implants. These alterations contribute towards the mechanism for infertility due to intraperitoneal exudate of unknown cause, even in the presence of normal ovulatory function. In the present study, we confirmed that the volume of peritoneal fluid had an inverse correlation with the disease stage in patients with endometriosis, whereas in the control group we often had to exclude patients from the study due to the absence of peritoneal fluid. This negative correlation has also been observed by others.^28^

Pittaway et al.^3^ evaluated the serum variations of CA-125 and observed that they corresponded to the surface involved by endometriosis, thus suggesting a 10- to 100-fold increase in the levels of this marker in peritoneal fluid. Progressive elevation of this marker has also been observed in perito-neal fluid, similar to that in serum.^14,17^ This feature was confirmed in the present study, in which peritoneal fluid CA-125 levels were 100- to 1000-fold higher than serum levels in women with pelvic endometriosis. We also observed a correlation between serum and peritoneal fluid CA-125 levels, thus suggesting peritoneal production and hematogenic absorption. Koninckx & Martin,^6^ after evaluating CA-125, concluded that superficial disease causes its elevation in peritoneal fluid, whereas deep disease causes its elevation in blood. We could not confirm these findings in our study, since CA-125 levels increased with deeper infiltration of the disease, both in blood and in peritoneal fluid, from women with pelvic endometriosis, in comparison with to normal patients. This confirmatory paper may help in understanding the physiopathology of this enigmatic disease and may be used for future meta-analysis, since there are several controversial points regarding CA-125 levels in endometriotic patients.

## CONCLUSION

We found high concentrations of CA-125 in peritoneal fluid from women with and without endometriosis, and there was a positive correlation between serum and peritoneal fluid CA-125 levels, thus suggesting that monitoring of CA-125 in peripheral blood may reflect its behavior in the abdominal environment. Moreover, we demonstrated that the levels of CA-125 in serum and peritoneal fluid were higher in women with endometriosis, but probably without diagnostic significance in detecting the disease.
